# Patient-reported reactogenicity and safety of COVID-19 vaccinations vs. comparator vaccinations: a comparative observational cohort study

**DOI:** 10.1186/s12916-023-03064-6

**Published:** 2023-09-19

**Authors:** Felix Werner, Nikoletta Zeschick, Thomas Kühlein, Philipp Steininger, Klaus Überla, Isabelle Kaiser, Maria Sebastião, Susann Hueber, Lisette Warkentin

**Affiliations:** 1 Institute of General Practice, Friedrich-Alexander-Universität Erlangen-Nürnberg (FAU), Uniklinikum Erlangen, Universitätsstraße 29, Erlangen, 91054 Germany; 2 Institute of Clinical and Molecular Virology, Friedrich-Alexander-Universität Erlangen-Nürnberg (FAU), Uniklinikum Erlangen, Schloßgarten 4, Erlangen, Germany; 3 Department of Medical Informatics, Biometry and Epidemiology, Friedrich-Alexander-Universität Erlangen-Nürnberg (FAU), Waldstraße 6, Erlangen, Germany

**Keywords:** COVID-19, SARS-CoV-2, Vaccine, Adverse drug reaction, Medical consultation, Observational study, Survey, Reactogenicity

## Abstract

**Background:**

In the course of the SARS-CoV-2 pandemic, multiple vaccines were developed. Little was known about reactogenicity and safety in comparison to established vaccines, e.g. influenza, pneumococcus, or herpes zoster. Therefore, the present study aimed to compare self-reported side effects in persons vaccinated against SARS-CoV-2 with the incidence of side effects in persons receiving one of the established vaccines.

**Methods:**

A longitudinal observational study was conducted over a total of 124 days using web-based surveys. Persons receiving either a vaccination against SARS-CoV-2 or one of the established vaccines (comparator group) were included. In the first questionnaire (short-term survey), 2 weeks after vaccination, mainly local and systemic complaints were evaluated. The long-term survey (42 days after vaccination) and follow-up survey (124 weeks after vaccination) focused on medical consultations for any reason. Multivariate analyses were conducted to determine the influence of the vaccine type (SARS-CoV-2 vs. comparator) and demographic factors.

**Results:**

In total, data from 16,636 participants were included. Self-reported reactogenicity was lowest in the comparator group (53.2%) and highest in the ChAdOx1 group (85.3%). Local reactions were reported most frequently after mRNA-1273 (73.9%) and systemic reactions mainly after vector-based vaccines (79.8%). Almost all SARS-CoV-2 vaccines showed increased odds of reporting local or systemic reactions. Approximately equal proportions of participants reported medical consultations. None in the comparator group suspected a link to vaccination, while this was true for just over one in 10 in the mRNA-1273 group. The multivariate analysis showed that people with SARS-CoV-2 vaccination were not more likely to report medical consultations; patients who had received a regimen with at least one ChAdOx1 were even less likely to report medical consultations. Younger age, female gender and higher comorbidity were mostly associated with higher odds of medical consultations.

**Conclusion:**

The rate of adverse reactions after established vaccinations was roughly comparable to previous studies. Two weeks after vaccination, participants in the SARS-CoV-2 vaccination group reported more local and systemic local reactions than participants in the comparator group. In the further course, however, there were no higher odds of medical consultations in either of the two groups. Thus, altogether, we assume comparable safety.

**Trial registration:**

DRKS-ID DRKS00025881 and DRKS-ID DRKS00025373.

**Supplementary Information:**

The online version contains supplementary material available at 10.1186/s12916-023-03064-6.

## Background

In December 2019, a novel viral infection associated with pneumonia as well as prominent general symptoms was reported for the first time in Wuhan, China [1]. Initially called the 2019 novel coronavirus (2019-nCoV), and later SARS-CoV-2, the infection spread rapidly across the globe. As a result of substantial efforts to expand research and development of vaccines against SARS-CoV-2, the first vaccine was already licensed in December 2020, followed by further vaccines on different pharmacological bases. Large randomised controlled trials have demonstrated the safety and efficacy of the vaccines BNT162b2 (Comirnaty®, INN Tozinameran from BioNTech and Pfizer), mRNA-1273 (Spikevax®, INN Elasomeran from Moderna), ChAdOx1 (Vaxzevria® from AstraZeneca and Oxford University) and Ad26.COV2.S (Jcovden® from Janssen-Cilag) [2–5]. Starting with the administration of BNT162b2 in December 2020, the overall vaccination rate in countries of the European Economic Area has reached 72.8% as per August 2022 [6]. One reason for the insufficient vaccination rate against SARS-CoV-2 is a more fundamental vaccination scepticism for various reasons, e.g. the fear of vaccination consequences [7], fuelled by a general scepticism due to the fast development, the novel technology of mRNA vaccines, and media reports of very rare adverse events such as *vaccine-induced immune thrombotic thrombocytopenia* [8] or myocarditis [9, 10]. This scepticism fell on fertile ground: While established vaccinations are considered highly effective in preventing diseases and are one of the most significant public health achievements of the modern era [11], vaccination hesitancy has been identified by the WHO as a key threat of global health [12]. In 2016, citizens in Europe were more critical of the safety and benefits of vaccination than citizens in other regions of the world, and seven of the ten countries with the least confidence in vaccine safety were located in Europe [13]. In 2020, after the rise of SARS-CoV-2 and before the development of corresponding vaccines, only 68% of German citizens reported to be willing to take a vaccine against SARS-CoV-2 even if it was proven safe and effective [14].

Against this background, the *Corona Vakzin Konsortium* (CoVaKo) project tries to elucidate the efficacy and safety of the SARS-CoV-2 vaccines under real-world conditions. The CoVaKo safety study reported here aimed to assess reactogenicity and self-reported health problems compared with other common vaccinations such as those against influenza or pneumococcus [15, 16]. While the incidence of adverse events after administration of established vaccines varies widely and severe consequences are exceedingly rare [11], a comparison of the new SARS-CoV-2 vaccines with the established vaccines is still lacking. The work presented aims to contribute to bridging this research gap by comparing patient-reported outcomes after vaccination with SARS-CoV-2 vaccines or established vaccines.

## Methods

We conducted a longitudinal online cohort observational study to assess health problems and healthcare utilisation after vaccination against SARS-CoV-2 and multiple other vaccinations. Reporting of the study is based on the STROBE (Strengthening the Reporting of Observational studies in Epidemiology) recommendations (see Additional file 1) [17].

### Study design and recruiting

Web-based surveys were used to elucidate reactogenicity and health problems that occurred within a total time of 124 days after the vaccinations and that have resulted in medical consultation, medication intake or sick leave. Vaccinations included SARS-CoV-2 (SARS-CoV-2 vaccine group) and influenza, pneumococcus, tick-borne encephalitis (TBE), tetanus and diphtheria (Td) vaccinations with or without pertussis and poliomyelitis (TdaP and TdaP-IPV), and herpes zoster (comparator group). Short- and long-term surveys were sent 14 and 40 days after vaccination, respectively. Additionally, participants received the follow-up survey 124 days after vaccination.

**Fig. 1:**
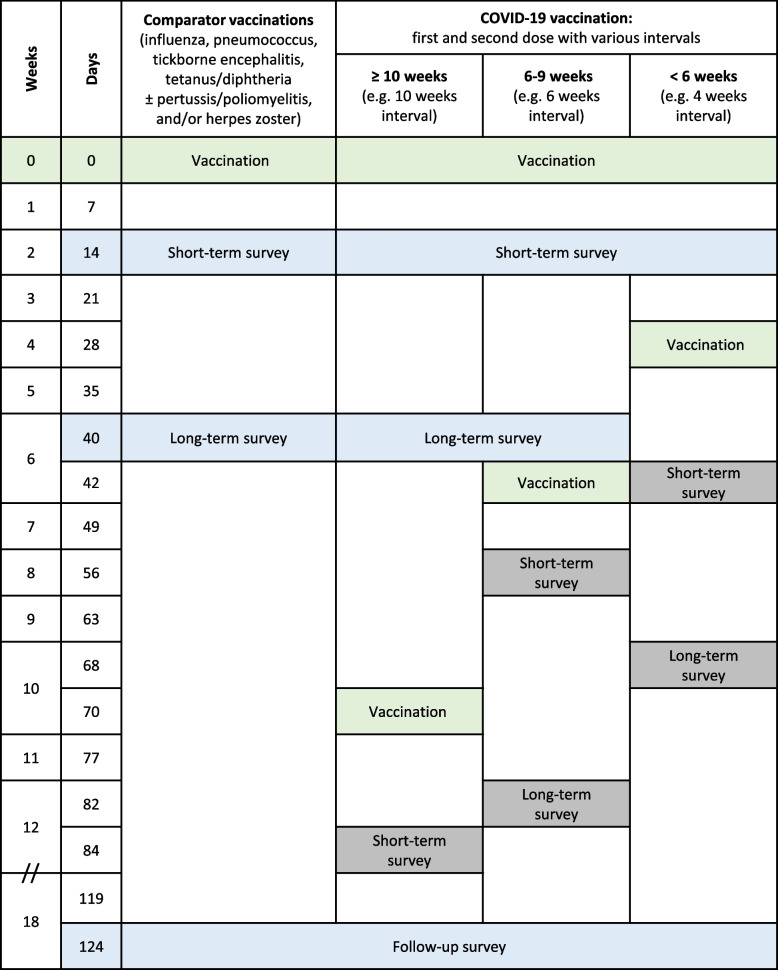
Depiction of the sequence of the survey dates. After vaccination against SARS-CoV-2 or against one of the comparators, participants received an invitation to the short-term survey at 14 days latency, and an invitation to the long-term survey after a further 26 days. One hundred twenty-four days after vaccination, all participants received an invitation to the follow-up survey. Surveys included in the analysis are highlighted in blue, while those excluded from the analysis are highlighted in grey

Due to the changing SARS-CoV-2 vaccination recommendations over time, the intervals between first and second vaccinations changed over the course of the study. It was therefore decided to omit individual survey time points when short- and long-term surveys were in an unfavourable temporal relationship. Figure 1 provides an overview of the sequence of survey time points for both groups and immunisation regimes.

The recruitment strategy and the surveys were evaluated in a feasibility study [18] (DRKS ID DRKS00025881 [19]). Recruitment started on April 17th, 2021 for the feasibility study and on May 20th, 2021 for the main study (DRKS ID DRKS00025373 [16]) in vaccination centres as well as primary care and occupational physicians’ practices in Bavaria, Germany. Recruitment continued until April 17th, 2022, data collection period closed on August 28th, 2022. After vaccination, leaflets were given to individuals providing information about the study. Individuals had the opportunity to voluntarily register on a secure web-based platform and give their informed consent. Participants should preferably register at the time of the first vaccination. However, registration was possible during the whole observation period. They were then provided via e-mail with links to the relevant surveys. Depending on the time of registration participants received links to the short-term, long-term and/or follow-up surveys. After receiving the link to the survey, participants had to respond within 5 days. Given the dynamic changes in COVID-19 vaccine schedules and the importance of obtaining real-world evidence on vaccine safety, data from both the feasibility study and the main study were included in the data analysis. This was considered to be a methodologically sound approach by the authors, as there was little change in the survey between the feasibility study and the main study. All survey methods were implemented in accordance with the relevant guidelines and regulations.

Sample size planning was conducted prior to the start of the study, assuming an event probability of 0.1% for rare events. The corresponding 95% confidence interval ranges from 0.02 to 0.29% for *N* = 3000 according to Clopper and Pearson [20]. For larger event probabilities, the width of the confidence interval narrows.

### Participants and data acquisition

Patients who were older than eleven years (older than 17 years at the time of the study start due to changing recommendations) and had received a vaccination against SARS-CoV-2, influenza, pneumococcus, tickborne encephalitis, tetanus with or without diphtheria/pertussis/poliomyelitis, and/or herpes zoster in the last 124 days were included. Exclusion criteria were incomplete registrations, registration before vaccination date or later than 124 days after vaccination of first or single dose, and an interval between prime and boost SARS-CoV-2 immunisation of less than 14 days.

At registration, participants were asked about socio-demographic characteristics, comorbidities and information about the vaccination including brand name and batch number. Morbidity was assessed based on a modified German version of the Self-Administered Comorbidity Questionnaire (m-SCQ-D) [21, 22]. In the short-term survey, solicited and unsolicited local and systemic reactions were recorded. The endpoint ‘local reactions’ is composed of pain, erythema or swelling, limitation of movement and abscess, while ‘systemic reactions’ includes headache, fatigue, nausea or vomiting, fever or chills, muscle or joint pain, allergic reactions, dyspnoea, syncope, seizure, dizziness, numbness or paraesthesia, and coagulation disorder. Unsolicited reactions were those reactions not covered by the prespecified local or systemic reactions and could be reported in a free text field. The participants were asked to specify any consequences of the observed reactions. These included medication intake, sick leave, ambulatory consultation, hospital outpatient consultation, or hospitalisation. In the long-term and follow-up surveys, participants were asked to report all health problems that led or will lead to consulting a doctor (outpatient consultation) or to seeking hospital care (inpatient consultation), including hospitalisation. The subjects were asked to report all health problems that occurred in the respective time interval and to assess afterwards whether they assumed an association with the vaccination and whether the health problem was pre-existing. All surveys can be found in the Additional file 2. If changes had to be made, mainly due to changes in vaccination recommendations, they are indicated in that document.

Data collection was carried out with the web-based software platform REDCap (Research Electronic Data Capture), hosted at Universitätsklinikum Erlangen [23, 24]. Data were recorded on a server of the Uniklinikum Erlangen.

### Data preparation

In case a person registered twice with the same email address, the data records were merged. If two participants used the same email address, both data sets were handled separately. For purposes of plausibility testing, it was checked whether the invitation links were sent at the correct time with regard to the vaccination date. If the answers were sent at an incorrect time, they were counted as missing. In case of implausible data on age (year of birth before 1900), weight (less than 30 kg or more than 300 kg), height (less than 100 cm or more than 250 cm) and/or pregnancy (for male participants or participants with year of birth before 1975), the corresponding variables were set to missing. Batch numbers were checked for plausibility and compared with the database of the Federal Institute for Drugs and Medical Devices (Bundesinstitut für Arzneimittel und Medizinprodukte). Data records with invalid batch numbers were excluded. The total number of completed questionnaires included in the analysis was 16,636.

The analysis compared the following cohorts:

- Short-term survey: BNT162b2, mRNA-1273, ChAdOx1, Ad26.COV2.S, and comparator vaccinations
- Long-term survey: BNT162b2, mRNA-1273, ChAdOx1, Ad26.COV2.S, and comparator vaccinations
- Follow-up survey: homologous mRNA (BNT162b2 + BNT162b2 or mRNA-1273 + mRNA-1273), homologous vector (ChAdOx1 + ChAdOx1), heterologous immunisation (ChAdOx1 + BNT162b2 or ChAdOx1 + mRNA-1273), and comparator vaccinations

**Fig. 2:**
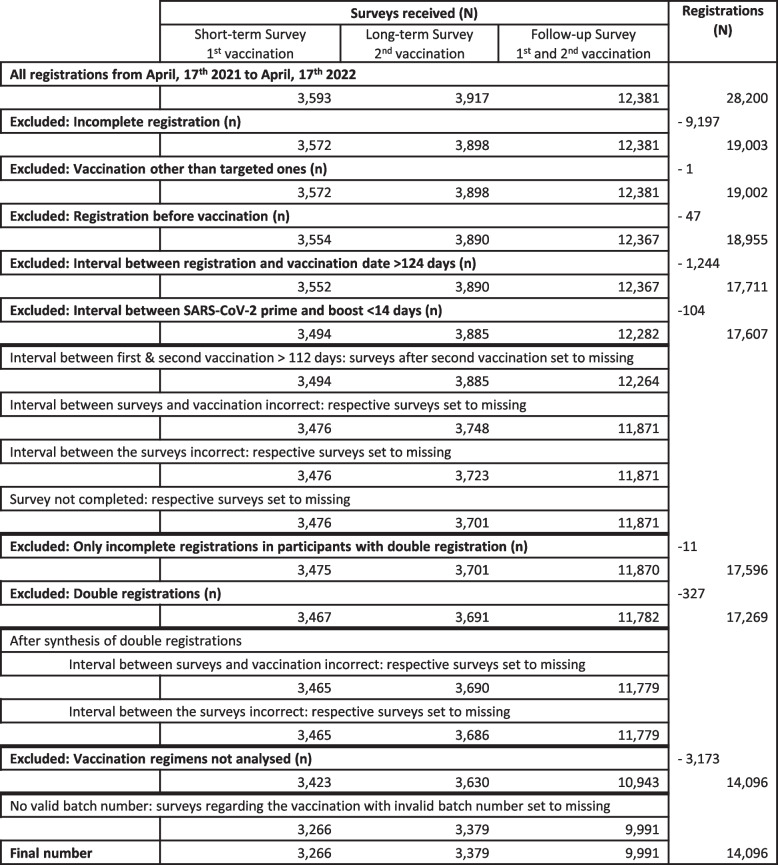
Data selection and preparation process. If a person registered twice with the same email address, data sets were merged. If an email address was used by more than one participant, data records were considered separately. Batch numbers were checked for plausibility: surveys regarding vaccinations with invalid batch numbers were counted as missing; invalid batch number was defined as an unknown number or an incorrect combination of number and vaccine

The data selection and preparation process are depicted in Fig. 2.

### Statistical analysis

Age was determined by subtracting the reported year of birth from the registration year (2021/2022). Sociodemographic characteristics and comorbidities are reported as proportion or as mean/median. Comorbidity in form of m-SCQ-D was calculated. Consequences of reactions were queried in a multiple-choice question. The consequence that is considered to be the most severe is being reported (hierarchically ordered from no consequence to medication intake, sick leave, ambulatory consultation, hospital outpatient consultation, and hospitalisation). Health problems are reported as absolute and relative frequencies.

To compare cohorts, multivariable logistic regressions were performed. For the short-term surveys, the outcome was local and systemic reactions. For the long-term and follow-up surveys, the outcomes were outpatient and inpatient medical consultations that had occurred or were planned. Predictors included vaccination type (short-term and long-term surveys) or vaccination regimen (follow-up survey), respectively, age (in years), gender, m-SCQ-D, vaccination-registration interval (in days), and another vaccination in the 8 weeks prior to registration or since answering the last survey. Reference was represented by comparator vaccinations. We conducted a subgroup analysis including only those participants who had received influenza vaccination in the reference group.

R Statistical Software (version 4.0.2, R Foundation for Statistical Computing, Vienna, Austria) was used for conducting data preparation, analyses, and figure creation.

## Results

### Sociodemographic characteristics

In total, 3266 short-term surveys, 3379 long-term surveys and 9991 follow-up surveys have been included in the analysis. In the short-term survey, the SARS-CoV-2 vaccination group comprised 3063 participants whereas the comparator group included 203 individuals (Table 1). All comparator vaccines were inactivated vaccines. In the SARS-CoV-2 vaccination cohort, most of the individuals had a vaccination with an mRNA vaccine; while vector-based vaccines were more likely to be given to male participants, mRNA vaccines were mostly administered to female participants. In the short-term survey, participants in the comparator group were to a larger extent female and had a higher mean age than those in the SARS-CoV-2 vaccination group (comparator group: 59.1% female, 49.8 years; SARS-CoV-2 group: 55.5% female, 42.0 years). The age difference was least prominent compared with participants who received ChAdOx1 (46.1 years) and most pronounced compared to participants vaccinated with Ad26.COV2.S (39.2 years). Participants in the comparator group more frequently reported no pre-existing diseases than participants with a SARS-CoV-2-vaccination (37.4% vs. 44.7 to 60.0% for short-term surveys, 33.5% vs. 40.0 to 61.0% for long-term surveys and 32.8% vs. 29.9 to 41.9% for follow-up surveys). Body mass index was similar between the groups. In the comparator cohort, around one-third reported having received another immunisation in the 8 weeks before receiving the vaccination.

**Table 1:** Sociodemographic characteristics of participants who responded to the surveys

	Short-term survey					Long-term survey					Follow-up survey					
	mRNA		Vector		Comp	mRNA		Vector		Comp	Homol. mRNA		Homol. vector	Heterol.		Comp
	BNT	MOD	AZ	JAN		BNT	MOD	AZ	JAN		BNT BNT	MOD MOD	AZ AZ	AZ BNT	AZ MOD	
***N***	2044	687	177	155	203	2002	689	192	159	337	5539	1342	471	1382	879	378
**Gender** (%)																
Female	55.9	62.6	36.7	41.3	59.1	56.0	61.4	37.0	42.1	58.2	58.8	61.2	41.0	61.9	60.4	58.2
Male	44.0	37.1	63.3	58.7	40.4	44.0	38.5	63.0	57.9	41.5	41.1	38.5	59.0	38.1	39.5	41.5
Diverse	0.0	0.3	0.0	0.0	0.5	0.0	0.1	0.0	0.0	0.3	0.1	0.4	0.0	0.0	0.1	0.3
**Age**																
Mean	42.1	41.4	46.1	39.2	49.8	44.6	41.7	48.4	39.7	50.1	45.6	44.6	57.3	48.9	48.4	50.8
SD	14.8	14.4	15.9	12.9	14.8	15.3	14.4	16.2	12.9	14.5	15.2	14.3	15.4	13.6	14.3	14.5
**No pre-existing diseases** (%)																
	44.8	44.7	48.0	60.0	37.4	40.0	44.1	42.2	61.0	33.5	38.9	39.9	29.9	37.4	41.9	32.8
**m-SCQ-D**																
Median	0	0	0	0	1	0	0	0	0	1	0	0	0	0	0	1
IQR	0–2	0–2	0–2	0–2	0–2	0–2	0–2	0–2	0–2	0–2	0–2	0–2	0–2	0–2	0–2	0–2
**BMI**																
Mean	25.4	25.7	25.2	25.1	26.1	25.8	25.8	25.6	25.2	26.0	25.8	25.9	26.8	25.6	25.9	25.8
SD	5.4	5.4	4.3	4.0	6.1	5.6	5.4	4.6	4.2	5.8	5.4	5.4	5.3	4.9	5.2	5.7
NA	27	16	3	1	4	28	13	2	1	7	59	21	6	14	1	6
**Participants with other vaccinations 8 weeks before first vaccination** (%)																
	5.8	4.8	7.9	3.2	31.6	5.7	4.6	7.3	3.1	31.8	7.2	4.8	5.1	6.2	7.3	32.6

*BNT* BNT162b2, *MOD* mRNA-1273, *AZ* ChAdOx1, *JAN* Ad26.COV2.S, *m-SCQ-D* modified German version of the Self-Administered Comorbidity Questionnaire, *BMI* body mass index, *NA* not available

### Descriptive results of the short-term survey

Considerably more participants in the SARS-CoV-2 vaccine group reported at least one local, systemic or unsolicited reaction than participants in the comparator cohort (local: 47.7% to 73.9% vs. 42.9%, systemic: 48.5% to 81.9% vs. 32.5%, unsolicited: 9.7% to 15.8% vs. 5.4%; Table 2). In the case of vector-based vaccines, local reactions were reported less frequently than systemic reactions (51.2% vs. 79.8%). Participants reported more local than systemic reactions for the mRNA-based vaccines (61.8% vs. 51.4%) and the comparator vaccines (42.9% vs. 32.5%).

**Table 2:** Symptom-burden within 14 days after the first vaccination (short-term survey)

	Short-term survey after first vaccination				
***N***	mRNA		Vector		Comparator
	BNT	MOD	AZ	JAN	
	2044	687	177	155	203
**At least one symptom** (*N* (%))					
	1437 (70.3)	570 (83.0)	151 (85.3)	125 (80.6)	108 (53.2)
**At least one local reaction** (*N* (%))					
	1175 (57.5)	508 (73.9)	96 (54.2)	74 (47.7)	87 (42.9)
Consequences of local reactions (%)					
No consequences	95.0	86.2	94.8	90.5	97.7
Medication intake	3.0	8.5	4.2	6.8	2.3
Sick leave	1.0	3.0	1.0	1.4	0.0
Ambulatory consultation	1.0	2.0	0.0	1.4	0.0
Hospital outpatient consultation	0.0	0.4	0.0	0.0	0.0
Hospitalisation	0.0	0.0	0.0	0.0	0.0
**At least one systemic reaction** (*N* (%))					
	992 (48.5)	407 (59.2)	145 (81.9)	120 (77.4)	66 (32.5)
Consequences of systemic reactions (%)					
No consequences	71.3	67.1	46.2	40.8	81.8
Medication intake	19.5	21.6	37.9	33.3	13.6
Sick leave	4.5	5.4	11.0	23.3	3.0
Ambulatory consultation	3.8	5.9	4.8	2.5	1.5
Hospital outpatient consultation	0.6	0.0	0.0	0.0	0.0
Hospitalisation	0.3	0.0	0.0	0.0	0.0
**At least one unsolicited reaction** (*N* (%))					
	198 (9.7)	95 (13.8)	28 (15.8)	17 (11.0)	11 (5.4)
Consequences of unsolicited reactions (%)					
No consequences	67.2	72.6	75.0	52.9	45.5
Medication intake	14.6	10.5	10.7	23.5	9.1
Sick leave	6.1	2.1	3.6	23.5	27.3
Ambulatory consultation	11.1	14.7	7.1	0.0	2.0
Hospital outpatient consultation	0.5	0.0	3.6	0.0	0.0
Hospitalisation	0.5	0.0	0.0	0.0	0.0

Percentage and absolute numbers of participants with vaccinations against SARS-CoV-2 and comparator vaccinations who reported local or systemic reactions, each with their respective consequences. Multiple answers were possible. The consequence perceived as most serious is reported (from no consequence to medication intake, sick leave, ambulatory consultation, hospital outpatient consultation, and hospitalisation)

*BNT* BNT162b2, *MOD* mRNA-1273, *AZ* ChAdOx1, *JAN* Ad26.COV2.S

#### Local reactions

For most of the individuals, local vaccine reactions had no consequence (86.2% to 97.7%). If one was reported, medication intake was most frequent, ranging from 2.3% in the comparator group up to 8.5% in the mRNA-1273 group. Sick leave and ambulatory consultation were less frequent consequences (comparator group: 0.0% for both; mRNA-1273 group: 3.0% and 2.0%, respectively). Hospital outpatient consultation only occurred very rarely and only in participants with mRNA-1273. Hospitalisations due to local reactions were reported in none of the groups.

*Systemic reactions* entailed more consequences than local reactions. While the majority of participants in the comparator cohort (81.8%) and in the mRNA cohorts (71.3%/67.1%) as well reported no consequences, this proportion was markedly lower in those vaccinated with vector-based vaccines (46.2%/40.8%). In all groups, medication intake was the most common consequence but with distinct differences, being lowest in comparator vaccines (13.6%) and highest in vector-based vaccines (33.3%/37.9%). In a similar manner, sick leave was reported more frequently in the vector-based cohorts (11.0%/23.3%) than in the mRNA (4.5%/5.4%) or comparator (3.0%) cohorts. Ambulatory consultations occurred roughly in equally low frequencies in all groups, with a minimum in comparator-vaccinated participants. Hospital outpatient consultations and hospitalisations due to systemic reactions were only reported by participants vaccinated with BNT162b2 and were very rare events.

*Unsolicited reactions* were reported more frequently in patients with vaccinations against SARS-CoV-2 than those with comparator vaccinations (11.0% vs. 5.4%). For participants with comparator vaccinations, however, these had consequences more frequently in contrast to participants with SARS-CoV-2 vaccinations, mainly driven by taking sick leave.

### Multivariate regression analyses of local and systemic reactions reported in the short-term surveys

#### Local reactions

In contrast to the comparator group, participants in the SARS-CoV-2 vaccination group showed higher odds of reporting any, local, or systemic reactions (Table 3). Vaccination with mRNA-1273 was associated with particularly higher odds of reporting a local reaction (OR 3.12, 95% CI [2.20, 4.44]). Vaccination with BNT162b2 (OR 1.52, 95% CI [1.11, 2.09]) and ChAdOx1 (OR 1.69, 95% CI [1.10, 2.60]) showed smaller odds. There was no significant difference between the Ad26.COV2.S group and the comparator group. Younger age and female gender were associated with a higher reporting of local reactions. m-SCQ-D, interval between vaccination and registration, and receiving another vaccination had no influence on reporting.

**Table 3:** Multivariate regression analyses of local and systemic reactions in the short-term survey and of outpatient and inpatient consultations reported in the long-term and follow-up surveys. Odds ratio of reporting local and systemic adverse events in the short-term survey (A) and consulting a doctor (outpatient consultation) or seeking hospital care (inpatient consultation) (both occurred and planned) in the long-term (B) and follow-up survey (C) depending on the vaccine used compared to comparator vaccines, age, sex, comorbidities and interval between vaccination and study registration

**A**	**Short-term survey**							
	**Local reactions**				**Systematic reactions**			
***n* = 3262; reference = comparator vaccines**								
	**OR**	**95% CI**		***p*-value**	**OR**	**95% CI**		***p*-value**
		LL	UL			LL	UL	
Intercept	1.81	1.20	2.74	0.005	0.94	0.61	1.43	0.765
BNT	1.52	1.11	2.09	0.009	1.82	1.31	2.54	0.0004
MOD	3.12	2.20	4.44	< 0.0001	2.69	1.89	3.85	< 0.0001
AZ	1.69	1.10	2.60	0.017	11.52	7.04	19.29	< 0.0001
JAN	1.04	0.66	1.64	0.857	7.61	4.64	12.72	< 0.0001
Age	0.97	0.97	0.98	< 0.0001	0.97	0.97	0.98	< 0.0001
Female	2.13	1.84	2.48	< 0.0001	1.81	1.56	2.10	< 0.0001
m-SCQ-D	1.05	1.00	1.10	0.065	1.18	1.12	1.24	< 0.0001
Interval V-R	1.01	1.00	1.03	0.347	1.03	1.01	1.05	0.013
Another V	0.83	0.63	1.09	0.172	0.87	0.66	1.14	0.304
**B**	**Long-term survey**							
	**Outpatient consultation**				**Inpatient consultation**			
***n* = 3374; reference = comparator vaccines**								
	**OR**	**95% CI**		***p*-value**	**OR**	**95% CI**		***p*-value**
		LL	UL			LL	UL	
Intercept	0.14	0.08	0.23	< 0.0001	0.04	0.01	0.14	< 0.0001
BNT	0.87	0.61	1.25	0.436	0.54	0.25	1.26	0.128
MOD	0.98	0.66	1.49	0.936	0.52	0.20	1.41	0.190
AZ	1.42	0.84	2.48	0.183	1.14	0.33	3.52	0.829
JAN	1.43	0.81	2.48	0.208	0.74	0.16	2.73	0.675
Age	0.99	0.98	1.00	0.005	0.98	0.96	1.00	0.019
Female	1.58	1.28	1.96	< 0.0001	1.29	0.76	2.25	0.354
m-SCQ-D	1.27	1.21	1.34	< 0.0001	1.27	1.13	1.40	< 0.0001
Interval V-R	1.01	1.00	1.01	0.170	1.01	0.99	1.03	0.173
Another V	0.85	0.65	1.09	0.206	0.84	0.42	1.58	0.602
**C**	**Follow-up survey**							
	**Outpatient consultation**				**Inpatient consultation**			
***n* = 9978; reference = comparator vaccines**								
	**OR**	**95% CI**		***p*-value**	**OR**	**95% CI**		***p*-value**
		LL	UL			LL	UL	
Intercept	0.17	0.12	0.25	< 0.0001	0.02	0.01	0.04	< 0.0001
BNT + BNT	0.84	0.63	1.13	0.239	0.97	0.48	2.19	0.943
MOD + MOD	0.88	0.65	1.21	0.441	0.92	0.43	2.18	0.847
AZ + AZ	0.52	0.35	0.76	0.001	0.52	0.20	1.40	0.186
AZ + BNT	0.57	0.41	0.79	0.001	0.34	0.14	0.86	0.018
AZ + MOD	0.47	0.33	0.67	0.0001	0.24	0.09	0.65	0.005
Age	1.00	0.99	1.00	0.056	0.99	0.99	1.00	0.277
Female	1.38	1.24	1.54	< 0.0001	0.84	0.65	1.10	0.207
m-SCQ-D	1.26	1.22	1.29	< 0.0001	1.21	1.15	1.28	< 0.0001
Interval V-R	1.01	1.01	1.01	< 0.0001	1.01	1.01	1.02	< 0.0001
Another V	0.90	0.77	1.05	0.201	1.03	0.69	1.50	0.873

*BNT* BNT162b2, *MOD* mRNA-1273, *AZ* ChAdOx1, *JAN* Ad26.COV2.S, *OR* odds ratio, *95% CI* 95% confidence interval, LL lower limit, *UL* upper limit, *Interval V-R* interval between vaccination and study registration, *Another V* another vaccination in the 8 weeks prior to registration or since answering the last survey

Reference vaccination: comparator vaccination; sex reference: male; age in years; m-SCQ-D: continuous variable

#### Systemic reactions

In the case of systemic reactions, the odds of reporting were highly increased in participants with ChAdOx1 (OR 11.52, 95% CI [7.04, 19.29]) and Ad26.COV2.S (OR 7.61, 95% CI [4.64, 12.72]). mRNA-1273 (OR 2.69, 95% CI [1.89, 3.85]) and BNT162b2 (OR 1.82, 95% CI [1.31, 2.54]) both increased reporting significantly, but on a lower level. Younger age and female gender were as well associated with an increased reporting of systemic reactions. Lower m-SCQ-D and shorter interval between vaccination and registration were significantly associated with lower odds of reporting systemic reactions. Having another vaccination had no influence on reporting systemic reactions.

The subgroup analysis, which restricted the comparator group to influenza vaccinations, showed no relevant changes in the predictors (data not shown). However, we observed that participants with Ad26.COV2.S vaccine reported local reactions significantly more often than people with influenza vaccination (OR 1.78, 95% CI [1.09, 2.94]).

### Descriptive results the long-term and follow-up surveys

In both the long-term and the follow-up survey, approximately equal parts of participants reported having sought medical consultation (14.2% vs. 13.0 to 16.7% for long-term surveys and 20.9% vs. 16.4 to 21.6% for follow-up surveys). The proportion of participants who had sought medical consultation, declared that their health problems were unknown and that they suspected a connection with the vaccination was lowest in the comparison group (2.1% vs. 3.1 to 9.4% for long-term surveys and 0.0% vs. 2.3 to 4.2% for follow-up surveys) (Table 4). In the long-term survey, patients in the comparator group were less likely to report medical consultation due to musculoskeletal complaints (25% vs. 34 to 49% of patients with medical consultation). Cardiovascular complaints were reported least frequently in all groups in both surveys (9% to 19% of patients with medical consultation).

**Table 4:** Medical consultations, health problems and regarding patients’ views within 40 and 124 days after vaccination (long-term and follow-up surveys)

	Long-term survey after first vaccination					Follow-up survey after first vaccination					
	mRNA		Vector		Comp	Homologous mRNA		Homologous vector	Heterologous		Comp
	BNT	MOD	AZ	JAN		BNT BNT	MOD MOD	AZ AZ	AZ BNT	AZ MOD	
***N***	2002	689	192	159	337	5539	1342	471	1382	879	378
**Medical consultations** (%)											
Outpatient	11.0	11.2	15.1	12.0	11.3	18.4	18.3	15.9	15.8	14.0	19.8
Outpatient in planning	1.8	2.5	1.0	4.4	2.7	2.6	3.0	2.1	2.5	2.2	1.1
Inpatient	1.6	1.3	2.6	0.6	2.4	2.5	2.2	2.5	1.1	1.1	2.1
Inpatient in planning	0.0	0.1	0.0	1.3	0.3	0.4	0.3	0.2	0.4	0.0	0.3
**Participants with at least one medical consultation**											
%	13.0	13.8	16.7	16.5	14.2	21.4	21.6	18.3	18.4	16.4	20.9
*n*	261	95	32	26	48	1183	290	86	254	144	79
**thereof:**											
**HPlMC was unknown to the participant prior to vaccination (%)**											
All HPlMC	48.7	45.3	68.8	46.2	52.1	51.8	54.1	43.0	49.2	56.9	54.4
At least one HPlMC	35.2	35.8	31.2	38.5	33.3	34.5	32.4	36.0	34.3	28.5	34.2
**Participant suspected association of HPlMC to vaccination** (%)											
Regarding all HPlMC	5.0	12.6	3.1	7.7	2.1	2.9	3.8	3.5	2.8	4.2	0.0
Regarding at least one HPlMC	23.0	17.9	18.8	23.1	6.2	17.7	19.7	10.5	16.1	18.1	8.9
**All HPlMC unknown and association with vaccination is suspected by the participant** (%)											
	4.2	9.4	3.1	7.6	2.1	2.4	2.8	2.3	2.4	4.2	0.0
**Health problems leading to medical consultation** (%)											
Musculoskeletal disorders	36.8	49.5	34.3	41.9	25.0	40.3	38.3	48.7	38.9	38.2	39.2
General symptoms	42.5	52.6	31.2	38.1	50.0	41.9	46.9	29.0	31.5	36.1	40.5
Neurological disorders	36.4	44.2	37.4	34.3	29.2	29.2	30.7	26.7	26.0	29.8	29.1
Cardiovascular disorders	19.2	9.5	18.7	15.2	12.5	15.7	14.1	16.2	10.2	13.2	11.4
Unsolicited health problems	52.5	55.8	56.1	45.7	56.3	48.9	48.0	54.5	46.8	56.9	57.0
No health problem named	6.5	6.3	6.2	3.8	6.3	4.7	4.1	3.5	6.7	4.2	2.5

Percentage of participants with vaccinations against SARS-CoV-2 and comparator vaccinations who reported planned or occurred medical consultations; multiple answers were possible. Participants who reported at least one medical consultation were asked whether they were aware of the health problem before vaccination and whether they suspected a link to vaccination. In addition, the participants were asked to assign their health problems to a symptom complex; multiple answers were possible

*BNT* BNT162b2, *MOD* mRNA-1273, *AZ* ChAdOx1, *JAN* Ad26.COV2.S, *HPlMC* health problem leading to medical consultation

### Multivariate regression analyses of medical consultations and health problems reported in the long-term and follow-up surveys

#### Long-term survey

There was no statistically significant difference in out- or inpatient medical consultations with regard to vaccination (Table 3). Lower age, female gender, and higher m-SCQ-D were associated with higher odds of reporting an outpatient medical consultation. In the case of inpatient consultation, there was no influence of gender.

#### Follow-up survey

Odds of reporting medical out- or inpatient consultations was not higher in participants with homologous vaccination with BNT162b2 or mRNA-1273 than in the comparator group. However, participants with homologous vaccination with ChAdOx1 or heterologous vaccination reported significantly less outpatient medical consultations (homologous: OR 0.52, 95% CI [0.35, 0.76], ChAdOx1 + BNT162b2: OR 0.57, 95% CI [0.41, 0.79]; ChAdOx1 + mRNA-1273: OR 0.47, 95% CI [0.33, 0.67]). Female gender, higher m-SCQ-D, and longer interval between vaccination and registration was associated with more reports of outpatient consultations. Age had no influence on reporting outpatient consultations. In a similar manner, fewer inpatient consultations were reported by patients with heterologous vaccination, but not with ChAdOx1 homologous immunisation (ChAdOx1 + BNT162b2: OR 0.34, 95% CI [0.14, 0.86]; ChAdOx1 + mRNA-1273: OR 0.24, 95% CI [0.09, 0.65]). Age and gender had no influence on reporting of inpatient consultations, while the influence of m-SCQ-D and interval to registration persisted. Receiving another vaccination in the 2 months prior to registration or since answering the last survey had no influence on reporting out- or inpatient consultations in the long-term as well as in the follow-up surveys.

Restricting the reference group to only those participants who had received influenza vaccination did not lead to relevant changes in the results of the long-term and follow-up survey (data not shown). However, the results showed that participants with homologous ChAdOx1 vaccination — in line with the results for participants with heterologous vaccination in the main analysis — were significantly less likely to report inpatient consultations in the follow-up survey than people with influenza vaccination (OR 0.36, 95% CI [0.14, 0.96]).

## Discussion

A longitudinal observational study was conducted to contrast reactogenicity and medical consultations following SARS-CoV-2 and comparator vaccinations. In the comparator group, local and systemic reactions were less frequently reported. For both local and systemic reactions, those vaccinated with comparator vaccinations were more likely to report no consequences. If consequences were reported, medication intake and sick leave were most commonly reported in all groups. Multivariate regression analyses showed a strong influence of younger age, female gender, and more comorbidities on reporting local as well as systemic reactions. In the long-term survey and the follow-up survey, a comparable frequency of seeking medical consultation in all groups has been observed. Comparator-vaccinated patients were significantly less likely to suspect an association with the vaccination than patients who had received SARS-CoV-2 vaccination. In the follow-up survey, participants vaccinated with a regime including ChAdOx1 were less likely to report out- or inpatient medical consultations than participants in the comparator group.

Published data on side effects of the comparator vaccines differ strongly and are of limited comparability, e.g. due to the different approval status of sera in different countries, but also due to different survey designs, outcomes or study populations. Low incidences of local side effects in up to half of all patients have been reported after administration of different vaccines against influenza, FSME-Immun® (TBE), and Encepur® (TBE), with reports ranging between 6.7 and 44.7% of patients [25–28]. In a second group of vaccinations — Shingrix® (herpes zoster), Pneumovax® 23 (pneumococcus), Boostrix® (Td), and Boostrix-Polio® (TdaP-IPV) — a higher incidence of local reactions has been found (75.9 to 88.0%) [29–33]. Systemic adverse events show a similar distribution but on a lower level: Vaccinations against influenza and TBE remain a group with a low frequency of systemic side effects, ranging from 0.6 to 31.0% [25–28]. This contrasts with vaccinations with Shingrix®, against pneumococcus, and against tetanus and diphtheria (with and without pertussis or poliomyelitis) as a group with a high frequency of systemic side effects, ranging from 64.8 to 82.1% [29–33]. The reactogenicity reported in our study was roughly the same as observed in other studies. Similarly, the prevalence of systemic reactions was lower than the prevalence of local reactions. The SARS-CoV-2 vaccination group reported considerably higher prevalences of local as well as systemic reactions (SARS-CoV-2 vaccination group: 60.7 and 54.5%, respectively). However, higher reporting of adverse events after SARS-CoV-2 vaccination may partly also be attributed to a nocebo effect [34, 35]. This hypothesis is supported by corresponding results of recent research from New Zealand, in which media reports of vaccine-induced myocarditis led to increased reports of cardiac symptoms [36].

To our knowledge, consequences in the form of medication intake, sick leave, or medical consultations had rather been included as outcomes of the vaccination in vaccine studies before SARS-CoV-2 than as side effects of the vaccinations. Very few studies discussed these consequences as reactions to the vaccination itself, such as Nichol et al. who reported a work loss due to side effects of a influenza vaccination in healthy subjects of 2 days per 100 persons [37]. Seeking medical consultation due to vaccination side effects was a common consequence, with 4.7 to 7.6% of patients with vaccination against influenza, pneumococcus or tetanus and diphtheria reporting consultations [32, 38]. With the introduction of the SARS-CoV-2 vaccinations, the consequences of adverse events after vaccination were increasingly surveyed. In two German studies, 13.1 and 28.4% of participants reported taking at least 1 day of sick leave after the first and the second vaccination against SARS-CoV-2, respectively [39], and 8% of participants felt unable to work after the first vaccination with BNT162b2, increasing to 35% of participants after the second vaccination [40]. 5.6% of BNT162b2 vaccinees with at least one side effect reported a need for medical care [41]. We found considerably lower reports of consequences after SARS-CoV-2 vaccination, with consequences after comparator vaccinations even lower. In our study, no medical consultations due to local reactions were reported in the comparator group, and systemic reactions resulted in only 1.5% of that population reporting outpatient medical consultations. That proportion of outpatient consultations due to adverse events was slightly higher in participants with vaccination against SARS-CoV-2, with up to 2.0% for local reactions and up to 5.9% for systemic reactions. A similar pattern was also found for the medication intake and sick leave.

Female gender had a significant influence on reports of outpatient medical consultations, but not of inpatient medical consultations. Gender-related differences in consultation frequency have already been described and discussed several times and contrast different health behaviours in similar symptoms on one side with different symptom compositions on the other [42–44]. One possible explanation for the pattern we observed may be a filtering function of primary care — while women presented more often as outpatients, they might have been referred to inpatient care just as often as men. This explanation assumes that women were more likely to present to outpatient care due to symptoms—which is supported by our short-term data—but were not more likely to have severe symptoms requiring inpatient care than men. A higher prevalence of adverse events in women has been reported before in vaccinations against SARS-CoV-2 [45, 46] as well as in the vaccines used as comparators [47–50]. Despite the fact that antibody responses to specific vaccine doses often tend to be higher in females than in males, low dose vaccination of comparator vaccines for women to reduce the prevalence of side effects has not been systematically investigated or deliberated to our knowledge so far [51]. Yet, reduction of the vaccination dose against SARS-CoV-2 was discussed shortly after the start of the vaccination campaign and the observed increased side effect rate in women [52].

## Limitations and strengths

Due to the design as a prospective observational study, group sizes differed significantly. While 3063 participants responded to the first survey with SARS-CoV-2 vaccination, this number increased to a total of 9613 participants in the follow-up survey. For the comparator vaccinations, by contrast, responses were only by 203 (short-term survey), 337 (long-term survey) and 378 participants (follow-up survey), respectively. This pronounced difference makes comparisons of adverse events with a very low incidence particularly difficult, even more so because the survey was powered for an event rate of 0.1%. Therefore, we performed a multivariate logistic regression to consider the different vaccination recommendations with regard to gender and age, as descriptive results may not be transferable to the German population as a whole. Secondly, resulting from the limited number of participants who had received one of the comparator vaccinations, we opted to combine the corresponding group. Consequently, this group comprised above all, though not exclusively, adjuvanted inactivated vaccines for which greater reactogenicity has been reported [53]. Thus, we performed a subgroup analysis focusing on participants with influenza vaccination only. This analysis did not reveal significant changes in the odds ratios of reporting local or systemic reactions, as well as outpatient and inpatient consultations.

Participants in our survey tended to be younger than would be expected based on vaccination recommendations, presumably due to an age-related digital divide in the sense of a selection bias. As higher reactogenicity after vaccination against SARS-CoV-2 in younger patients has been reported before [54], we assume that we tend to overestimate the reactogenicity of some vaccinations. However, we could hardly find any data on age-related reactogenicity after other than SARS-CoV-2 vaccinations. We also consider the possibility of survivor bias, which means that very rare but fatal health problems may be underrepresented in our data.

One of the strengths of the survey is the reporting of adverse events by the participants themselves, i.e. patient-reported outcome measures. As the data basis does not consist of medical reports but of self-reports, this survey allows for a direct and unmediated comparison of the frequency of reactions and medical consultations. However, due to this direct reporting by patients, it must be considered that non-response bias might lead to a skewing of the results and are therefore subject to uncertainty.

## Conclusions

Participants in the SARS-CoV-2 vaccination group were more likely to report local and systemic side effects 14 days after their vaccination than those in the comparator group. However, 16 weeks later, there was no higher reporting of seeking medical consultations in the SARS-CoV-2 vaccination group. Patients who had received ChAdOx1 at least once even reported significantly fewer outpatient and inpatient consultations than the comparator group. Adverse events and medical consultations were reported significantly more frequently by women, younger people and people with pre-existing medical conditions.

### Supplementary Information

**Additional file 1.** STROBE.

**Additional file 2.** Surveys.

## Data Availability

Aggregated data that support the findings of this study are available from the corresponding author for researchers who provide a methodologically sound proposal after consent of the data protection supervisor.

## Abbreviations

Ad26.COV2.S: Jcovden® (Janssen-Cilag)
BMI: Body mass index
BNT162b2: Comirnaty® (INN Tozinameran; BioNTech/Pfizer)
ChAdOx1: Vaxzevria® (AstraZeneca/Oxford University)
CoVaKo: *Corona* *Vakzin* *Konsortium* (Corona Vaccine Consortium)
COVID-19: Coronavirus disease 2019
mRNA: Messenger ribonucleic acid
mRNA-1273: SpikeVax® (INN Elasomeran; Moderna)
m-SCQ-D: Self-Administered Comorbidity Questionnaire, modified German version
REDCap: Research Electronic Data Capture
SARS-CoV-2: Severe acute respiratory syndrome coronavirus type 2
STROBE: Strengthening the Reporting of Observational studies in Epidemiology
TBE: Tick-borne encephalitis
Td: Tetanus and diphtheria
TdaP(-IPV): Tetanus, diphtheria, pertussis and polio
WHO: World Health Organization
