# Supplementary material for: Patient-reported reactogenicity and safety of COVID-19 vaccinations vs. comparator vaccinations: a comparative observational cohort study
Source: BMC Med. 2023 Sep 19;21:358. doi: 10.1186/s12916-023-03064-6 (PMC10510262; doi:10.1186/s12916-023-03064-6)
Supplement: Supplementary file 2 — Additional file 2. Surveys. [file 12916_2023_3064_MOESM2_ESM.docx]

Surveys

[Information 2](#_Toc131765499)

[Registration 2](#_Toc131765500)

[Personal Details 2](#_Toc131765501)

[Vaccination 3](#_Toc131765502)

[Vaccination Details 5](#_Toc131765503)

[Morbidity 10](#_Toc131765504)

[Short-term survey (after First Vaccination) 11](#_Toc131765505)

[Vaccination 11](#_Toc131765506)

[Reactogenicity 12](#_Toc131765507)

[Consequences 14](#_Toc131765508)

[Perception 15](#_Toc131765509)

[Short-term survey after Second Vaccination 16](#_Toc131765510)

[Vaccination 16](#_Toc131765511)

[Reactogenicity 17](#_Toc131765512)

[Consequences 18](#_Toc131765513)

[Perception 19](#_Toc131765514)

[Long-term/Follow-up survey 21](#_Toc131765515)

[Vaccination 21](#_Toc131765516)

[Medical care 22](#_Toc131765517)

[Perception 25](#_Toc131765518)

[Medical report 25](#_Toc131765519)

Information

In the following you will find a translation of the questions of the surveys that we used to assess reactogenicity and safety of COVID-19 primary immunisation and booster vaccination regimens as well as other common vaccinations. The most important information for programming the surveys are included, however, it is not complete. If you are interested in the original (German) surveys, please contact the authors.

Registration

Personal Details

1. Please enter your e-mail address:
2. Please enter your year of birth:

*Added later:* Are you already 12 years old?

*Comment: Added due to changes in recommendations regarding age. Before the age limit was 18 years. Participants younger than 18 years had to complete the survey with their custodian.*

**PROG**: Allow only one response option.

| Yes | ⭘ | Continue to the next question |
| --- | --- | --- |
| No, I would like to end my participation. | ⭘ | **PROG**: **END** |

1. Please indicate your gender:

| Male | ⭘ |
| --- | --- |
| Female | ⭘ |
| Diverse | ⭘ |

1. *Added later:* Are you pregnant or are you nursing?

*Comment: Added due to changes in recommendations.*

| Yes | ⭘ |
| --- | --- |
| No | ⭘ |

1. Please state your height in cm:
2. Please state your weight in kg. Indicate in whole numbers:
3. Please select the highest level of education you have achieved so far:

**PROG**: Allow only single-choice answers.

| Still student (*Added later due to changes in age recommendations)* | ⭘ |
| --- | --- |
| No degree | ⭘ |
| Lower certificate | ⭘ |
| Intermediate certificate | ⭘ |
| Completed apprenticeship | ⭘ |
| High school diploma | ⭘ |
| University degree | ⭘ |
| Not specified | ⭘ |

1. Which employment situation applies best to you? Please note that employment means any paid activity or activity that is associated with an income.

**PROG**: Allow only single-choice answers.

| Employed | ⭘ |
| --- | --- |
| In education | ⭘ |
| Unemployed | ⭘ |
| Retired | ⭘ |
| Other | ⭘ |
| Not specified | ⭘ |

1. How many inhabitants does your place of residence count?

**PROG**: Allow only single-choice answers.

| Below 5.000 (rural area) | ⭘ |
| --- | --- |
| 5.000 to approx. 20.000 (small town) | ⭘ |
| 20.000 to approx. 100.000 (medium-sized town) | ⭘ |
| 100.000 or more (city) | ⭘ |
|  |  |

Vaccination

For the following questions you will need your vaccination certificate.

1. Against which of the following diseases/pathogens have you been vaccinated most recently?
   *Addition added later due to difficulties in comprehension:*

“The vaccination for which you obtained the flyer and/or your last vaccination. (Note: Please select "More than one of the mentioned vaccinations" for vaccinations received on the same day) “

**PROG**: Allow only single-choice answers.

| Influenza | ⭘ |
| --- | --- |
| Shingles | ⭘ |
| Pneumococcus | ⭘ |
| COVID-19  *Addition added later due to changes in recommendation:*  “(if applicable + a further vaccination)” | ⭘ |
| TBE | ⭘ |
| Tetanus (and/or diphtheria/pertussis/poliomyelitis) | ⭘ |
| Several of the ones mentioned above  *Addition added later due to changes in recommendation:*  “on the same day (not COVID-19)” | ⭘ |
| None of the above | ⭘ **PROG: END** |

1. Have you ever been vaccinated against _______ before?

**PROG**: Allow only single-choice answers. The vaccination selected in question 11 appears in the placeholder unless "more than one" was checked.

| Yes | ⭘ |
| --- | --- |
| No | ⭘ |
| I do not know | ⭘ |

1. Are you currently receiving only a single vaccination/dose against COVID-19?

*Addition added later due to changes in recommendation:*

“For example, due to a previous COVID-19 infection or you are receiving a Booster vaccination (3rd vaccination or 2nd vaccination after vaccination with Johnson&Johnson (Janssen)).”

**PROG**: Allow only single-choice answers. Only appears if “COVID-19” was selected in question 11.

| Yes | ⭘ |
| --- | --- |
| No | ⭘ |

1. *Added later:* Why do you only receive one vaccination against COVID-19?

*Comment:* *Added later due to changes in recommendations*

**PROG**: Allow only single-choice answers. Only appears if “Yes” was selected in question 13.

| Booster (third vaccination and/or second vaccination after vaccination with Johnson&Johnson (Janssen)) | ⭘ |
| --- | --- |
| COVID-19 infection | ⭘ |
| Intolerance of the vaccination | ⭘ |
| Other | ⭘ |

1. Against which of the following diseases/pathogens have you been vaccinated? Multiple-choice answers are possible.

**PROG**: Allow multiple-choice answers. Only appears if “Several …” was selected in question 11

| Influenza | ⭘ |
| --- | --- |
| Shingles | ⭘ |
| Pneumococcus | ⭘ |
| TBE | ⭘ |
| Tetanus (and/or diphtheria/pertussis/poliomyelitis) | ⭘ |

1. *Added later:* Did you receive a further vaccination on the day of your COVID-19 vaccination?

*Comment:* *Added later due to changes in recommendations*

**PROG**: Allow only single-choice answers. Only appears if “COVID-19” was selected in question 11.

| Yes | ⭘ |
| --- | --- |
| No | ⭘ |

1. *Added later:* Against which of the following diseases/agents have you been vaccinated?

*Comment:* *Added later due to changes in recommendations*

**PROG**: Allow multiple-choice answers. Only appears if “COVID-19” was selected in question 11 and “Yes” in question 16.

| Influenza | ⭘ |
| --- | --- |
| Shingles | ⭘ |
| Pneumococcus | ⭘ |
| TBD | ⭘ |
| Tetanus (and/or diphtheria/pertussis/poliomyelitis) | ⭘ |
| None of the above | ⭘ |

Vaccination Details

In the following you will be asked for the name of the vaccine. You will find this on the label in your vaccination certificate.

1. You received the following influenza vaccine:

**PROG**: Allow only single-choice answers. Only appears if “influenza” was selected. Free text field opens when “other” is checked.

| Afluria Tetra | ⭘ |
| --- | --- |
| Efluelda | ⭘ |
| Fluad Tetra | ⭘ |
| Flucelvax Tetra | ⭘ |
| Fluenz Tetra | ⭘ |
| Influsplit Tetra | ⭘ |
| Influvac Tetra | ⭘ |
| Influvac | ⭘ |
| Vaxigrip Tetra | ⭘ |
| Xanaflu Tetra | ⭘ |
| I do not know | ⭘ |
| Other | ⭘ |

1. You received the following shingles vaccine:

**PROG**: Allow only single-choice answers. Appears only if "shingles" was selected

| Shingrix | ⭘ |
| --- | --- |
| Zostavax | ⭘ |
| I do not know | ⭘ |

1. You received the following pneumococcal vaccine:

**PROG**: Allow only single-choice answers. Appears only if "pneumococcus" was selected

| Pneumococcal Polysaccharide Vaccine 23 | ⭘ |
| --- | --- |
| Pneumovax 23 | ⭘ |
| Prevenar 13 | ⭘ |
| I do not know | ⭘ |

1. You received the following TBD vaccine:

**PROG**: Allow only single-choice answers. Appears only if "TBD" was selected

| Encepur | ⭘ |
| --- | --- |
| FSMW-IMMUN | ⭘ |
| I do not know | ⭘ |

1. You received the following tetanus (and/or diphtheria/pertussis/poliomyelitis) vaccine:

**PROG**: Allow only single-choice answers. Appears only if "tetanus” was selected

| Boostrix (TdPa/TdPa-IPV) | ⭘ |
| --- | --- |
| Covaxis (TdPa) | ⭘ |
| Infanrix (DTPa) | ⭘ |
| Repevax (TdPa-IPV) | ⭘ |
| Revaxis (Td-IPV) | ⭘ |
| Td-IMMUN (Td) | ⭘ |
| Td Impfstoff Merieux® (Td) | ⭘ |
| Td-pur (Td) | ⭘ |
| TdaP-IMMUN (TdPa) | ⭘ |
| Tetanol pur | ⭘ |
| Tetanus-Impfstoff Mérieux® | ⭘ |
| Tetravac | ⭘ |
| I do not know | ⭘ |

1. *Added later:* You received the following COVID-19 vaccine as your first vaccination:

*Comment:* *Added later due to changes in recommendations*

**PROG**: Allow only single-choice answers. Only appears if “Booster” was selected in question 14.

| BNT162b2 (BioNTech/Pfizer) | ⭘ |
| --- | --- |
| mRNA-1273 (Moderna) | ⭘ |
| ChadOx1 (AstraZeneca) | ⭘ |
| Ad26.COV2.S (Johnson & Johnson) | ⭘ |

1. *Added later:* When did you receive your first vaccination?

*Comment:* *Added later due to changes in recommendations*

**PROG**: Only appears if “Booster” was selected in question 14.

1. *Added later:* You received the following COVID-19 vaccine as your second vaccination:

*Comment:* *Added later due to changes in recommendations*

**PROG**: Allow only single-choice answers. Only appears if “Booster” was selected in question 14.

| BNT162b2 (BioNTech/Pfizer) | ⭘ |
| --- | --- |
| mRNA-1273 (Moderna) | ⭘ |
| ChadOx1 (AstraZeneca) | ⭘ |
| Ad26.COV2.S (Johnson & Johnson) | ⭘ |
| No 2^nd^ vaccination (due to COVID-19 infection) | ⭘ |

1. *Added later:* When did you receive the second vaccination?

*Comment:* *Added later due to changes in recommendations*

**PROG**: Only appears if “Booster” was selected in question 14 and “No 2^nd^ vaccination” was not selected in question 24.

1. *Added later:* You received the following COVID-19 vaccine during your 3. vaccination:

*Comment:* *Added later due to changes in recommendations*

**PROG**: Allow only single-choice answers. Appears only if "Booster" was selected in question 14.

| BNT162b2 (BioNTech/Pfizer) | ⭘ |
| --- | --- |
| mRNA-1273 (Moderna) | ⭘ |
| ChadOx1 (AstraZeneca) | ⭘ |
| Ad26.COV2.S (Johnson & Johnson) | ⭘ |

1. You received the following COVID-19 vaccine:

*Addition added later due to changes in recommendation:*

*“*If you received two different vaccines, please refer to the first vaccination.”

**PROG**: Allow only single-choice answers. Appears only if “COVID-19” was selected in question 11 and “Booster” was NOT selected in question 14.

| BNT162b2 (BioNTech/Pfizer) | ⭘ |
| --- | --- |
| mRNA-1273 (Moderna) | ⭘ |
| ChadOx1 (AstraZeneca) | ⭘ |
| Ad26.COV2.S (Johnson & Johnson) | ⭘ |

1. When did you receive this/these (booster) vaccination(s)?

*Comment: “(booster)” has been added later due to changes in recommendations.*

**PROG**: Free text field. Appears if participant receives only one vaccination.

1. When did you receive the first vaccine dose?

*Addition added due to changes in recommendations:*

(If you register after the second vaccine, please still mention the date of the first COVID-19 vaccine. The date of the second vaccine will be asked in the following.)

**PROG**: Free text field. Appears only if participant receives two vaccine doses.

1. When did/will you receive the second vaccine dose?

**PROG**: Free text field. Appears only if participant receives two vaccine doses.

1. You received or will receive the following COVID-19 vaccine (second dose):

**PROG**: Allow only single-choice answers. Only appears if a date was set in question 32.

| BNT162b2 (BioNTech/Pfizer) | ⭘ |
| --- | --- |
| mRNA-1273 (Moderna) | ⭘ |
| ChadOx1 (AstraZeneca) | ⭘ |
| Ad26.COV2.S (Johnson & Johnson) | ⭘ |
| I do not know | ⭘ |

1. Please enter the batch number

*Addition added due to comprehensibility:*

(in case of COVID-19: first dose).

**PROG**: Free text field. Only appears if "None" or "Several …" was NOT selected in question 11 and “Booster” was NOT selected in question 14.

1. *Added later:* Please enter the batch number of your third COVID-19 vaccine/ booster.

*Comment:* *Added later due to changes in recommendations*

**PROG**: Free text field. Only appears if “Booster” was selected in question 14.

1. *Added later:* Please enter the batch number of your second COVID-19 vaccine/ dose.

*Comment:* *Added later due to changes in recommendations*

**PROG**: Free text field. Only appears if date of second vaccination is in the past or present.

1. Please enter your batch numbers: Enter batch numbers separated by commas.

**PROG**: Free text field. Only appears if "several" was selected in question 10.

1. *Added later:* Please enter the batch numbers of further vaccines:

*Comment:* *Added later due to changes in recommendations*

**PROG**: Free text field. Only appears if participant received further vaccines next to COVID-19

1. Did you receive another vaccination in the 8 weeks before your vaccination on [date entered]?

**PROG**: Allow only single-choice answers. Only appears if "COVID-19", "none" or "several" was not selected in question 11.

| Yes, against another disease | ⭘ |
| --- | --- |
| Yes, against the same disease | ⭘ |
| No | ⭘ |
| I do not know | ⭘ |

1. Against which diseases have you been vaccinated before then? (Multiple answers are possible)

**PROG**: Allow multiple-choice answers. Only appears if “Yes” was selected in question 39

| Influenza | ⭘ |
| --- | --- |
| Shingles | ⭘ |
| Pneumococcus | ⭘ |
| COVID-19 | ⭘ |
| TBE | ⭘ |
| Tetanus (and/or diphtheria/pertussis/poliomyelitis) | ⭘ |
| None of the above | ⭘ |

Morbidity

1. Please select the general health problems you suffer from. This does not include vaccination side effects. Please check the appropriate box. Multiple-choice answers are possible.

**PROG**: Multiple-choice answers are allowed.

| Heart disease | ⭘ |
| --- | --- |
| High blood pressure | ⭘ |
| Lung disease | ⭘ |
| Diabetes | ⭘ |
| Gastrointestinal problems | ⭘ |
| Kidney disease | ⭘ |
| Liver disease | ⭘ |
| Anemia | ⭘ |
| Coagulation disorders | ⭘ |
| Cancer | ⭘ |
| Depression | ⭘ |
| Ostheoarthritis | ⭘ |
| Back pain | ⭘ |
| Rheumatoide arthritis (other autoimmune disease) | ⭘ |
| Allergies | ⭘ |
| None pre-existing diseases | ⭘ |

1. Are you receiving treatment for the symptoms, taking medication or are you restricted in your daily activities?

**PROG**: Multiple-choice answers are allowed, except for the option “Neither”. The question appears for each health problem selected.

| Treatment/Medication | ⭘ |
| --- | --- |
| Restriction in daily life | ⭘ |
| Neither | ⭘ |

**End of registration.**

Short-term survey (after First Vaccination)

Thank you for participating in our study.

Mark the answer that most closely matches your assessment. There is no right or wrong.

Please click on "*Next Page*" to start the survey.

Vaccination

1. Did you receive another vaccination since the vaccination against [vaccination received]?

**PROG**: Allow only single-choice answers. The vaccination selected in the registration in appears in the placeholder.

| Yes | ⭘ |
| --- | --- |
| No | ⭘ |

1. You received the following vaccination(s) afterwards. Multiple-choice answers are possible.

**PROG**: Allow multiple response options. Only appears if "Yes" was selected in question 1.

| Influenza | ⭘ |
| --- | --- |
| Shingles | ⭘ |
| Pneumococcus | ⭘ |
| COVID-19 | ⭘ |
| TBE | ⭘ |
| Tetanus (and/or diphtheria/pertussis/poliomyelitis) | ⭘ |
| None of the above | ⭘ |

1. Have you been diagnosed with COVID-19 infection since your vaccination?

**PROG**: Allow only single-choice answers. Only appears if vaccination was against COVID-19.

| Yes | ⭘ |
| --- | --- |
| No | ⭘ |

1. Have there been any changes concerning your 2nd vaccination? Multiple-choice answers are possible.

*Comment: question has been replaced by the following due to changes in the recommendations. In the beginning the interval has been very strict, whereas later the interval changed more often.*

**PROG**: Multiple answer choices allowed. Only appears if "COVID-19" was selected in the registration.

| No changes | ⭘ |
| --- | --- |
| Yes, appointment cancelled | ⭘ |
| Yes, change of active substance | ⭘ |
| Other | ⭘ |

1. *Added later:* Is there still an appointment planned for your 2^nd^ vaccination?

*Comment: see comment above.*

**PROG**: Allow only single-choice answers.

| Yes | ⭘ |
| --- | --- |
| No | ⭘ |

1. *Added later:* Please enter the date for your 2nd vaccination.

*Comment: see comment above.*

**PROG**: Free text field or option “no date set yet”. Only appears if the date for the 2^nd^ vaccination has not been entered in the registration.

1. What was the reason for cancelling the appointment? Multiple-choice answers are possible.

**PROG**: Multiple answer options allowed. Appears only if there is no second vaccination anymore.

| COVID-19 infection after vaccination | ⭘ |
| --- | --- |
| Intolerance to vaccination | ⭘ |
| Other | ⭘ |

**PROG**: Free text field. Only appears if “Other” is selected in question 7.

1. You are to receive the following COVID-19 vaccine. You will find the name of the vaccine on the label in your vaccination certificate:

**PROG**: Allow only one response option. Appears only if in question 4 “Yes, change of active substance” was selected.

| BNT162b2 (BioNTech/Pfizer) | ⭘ |
| --- | --- |
| mRNA-1273 (Moderna) | ⭘ |
| ChadOx1 (AstraZeneca) | ⭘ |
| Ad26.COV2.S (Johnson & Johnson) | ⭘ |
| Not sure yet | ⭘ |

Reactogenicity

1. Did you experience any reactions since the ______ vaccination? Please also record reactions that you do not attribute to the vaccination.

**PROG**: Allow only single-choice answers. The placeholder shows the vaccination(s) selected in the registration.

| Yes | ⭘ |
| --- | --- |
| No | ⭘ **PROG**: **END** |

1. Which of the following complaints did you experience? Multiple-choice answers are possible.

**PROG**: Allow multiple-choice answers.

| Complaints at the injection site (e.g. swelling, redness, pain) | ⭘ |
| --- | --- |
| Restricted movement of the arm | ⭘ |
| Pus collection and/or abscess | ⭘ |
| Tiredness and/or fatigue | ⭘ |
| Fever > 38.0°C and/or chills | ⭘ |
| Nausea and/or vomiting | ⭘ |
| Allergic reaction (e.g. skin rash, facial swelling) | ⭘ |
| Shortness of breath | ⭘ |
| Headache | ⭘ |
| Sensory disturbance and/or numbness | ⭘ |
| Circulatory collapse | ⭘ |
| Dizziness | ⭘ |
| Seizure | ⭘ |
| Muscle or joint pain | ⭘ |
| Blood clotting disorder (e.g. thrombosis, embolism) | ⭘ |
| Other | ⭘ |

1. Which other complaints have you experienced? Please enter your other complaints here:

**PROG**: Free text field. Only appears if "Other" was selected.

1. Have you ever had an allergic reaction to a vaccine? (e.g. skin rash, shortness of breath, swelling in the throat or face, loss of consciousness).

**PROG**: Allow only single-choice answers. Only appears if "Allergic reaction", "Shortness of breath" or "Circulatory collapse" was selected in question.

| Yes | ⭘ |
| --- | --- |
| No | ⭘ |
| I do not know | ⭘ |

1. Has this been your first seizure?

**PROG**: Allow only single-choice answers. Only appears if “Seizure” was selected.

| Yes | ⭘ |
| --- | --- |
| No | ⭘ |
| I do not know | ⭘ |

Consequences

1. Which consequences did your health complaints have? Multiple-choice answers are possible.

**PROG**: Allow multiple-choice answers. Only appears if at least one reaction was selected. A separate question appears for each reaction selected.

| No consequences | ⭘ |
| --- | --- |
| Medication intake | ⭘ |
| Sick-leave | ⭘ |
| Outpatient (practice) consultation | ⭘ |
| Hospitalisation | ⭘ |
| Clinic (ambulant) consultation | ⭘ |

Please provide some information about your hospital stay.

1. How many times have you been to the hospital?

**PROG**: Allow only single-choice answers. Only appears if "Hospitalisation" and/or “Clinic (ambulant) consultation” was selected at least once.

| Once | ⭘ |
| --- | --- |
| Twice | ⭘ |
| 3 times | ⭘ |
| 4 times or more | ⭘ |

1. How long was your (longest) inpatient hospital stay in days?

**PROG**: Allow only single-choice answers. Only appears if " Hospitalisation" was selected at least once.

| 1 day | ⭘ |
| --- | --- |
| 2 days | ⭘ |
| 3 days | ⭘ |
| 4 days | ⭘ |
| 5 days | ⭘ |
| 6 days | ⭘ |
| 7 days | ⭘ |
| 8 days or more | ⭘ |

1. How did the admission(s) to the hospital take place?

**PROG**: Only appears if "Hospitalisation" and/or “Clinic (ambulant) consultation” was selected.

| Referral as a planned intervention by your general practitioner or specialist | ⭘ |
| --- | --- |
| Referral as an emergency by your general practitioner or specialist | ⭘ |
| Referral by a doctor on call | ⭘ |
| Transport by the ambulance service | ⭘ |
| Own presentation in the emergency room | ⭘ |

Perception

Please tell us how you felt about your health complaints.

**PROG**: Matrix fields.

1. I suspect a connection between my health complaints and the vaccination.
2. I feel/felt adversely affected by the health complaints.
3. My health complaints will have long-term consequences for me.
4. My health complaints are comparable to previous vaccinations.

**PROG**: Matrix fields. Allow only single-choice answers per question. Matrix only appears if at least one reaction has been selected.

| Strongly disagree | ⭘ |
| --- | --- |
| Disagree | ⭘ |
| Agree | ⭘ |
| Strongly agree | ⭘ |

1. With which complaints do you suspect a connection with the vaccination? Multiple-choice answers are possible.

**PROG**: Allow multiple-choice answers. Each reaction selected in question appears and can be checked.

**End of short-term survey**

Short-term survey after Second Vaccination

Thank you for taking part.

Mark the answer that most closely matches your assessment. There is no right or wrong.

Please click on "*Next Page*" to start the survey.

Vaccination

1. You received the following COVID-19 vaccine (2^nd^ dose). You will find the name of the vaccine on the label in your vaccination certificate:

**PROG**: Allow only one response option. Only appears if registration has been before second vaccination.

| BNT162b2 (BioNTech/Pfizer) | ⭘ |
| --- | --- |
| mRNA-1273 (Moderna) | ⭘ |
| ChadOx1 (AstraZeneca) | ⭘ |
| Ad26.COV2.S (Johnson & Johnson) | ⭘ |
| None, appointment cancelled | ⭘ |

1. *Added later:* Please enter the batch number of your 2. COVID-19 vaccine.

*Comment:* *In the beginning we only asked for the batch number of the first vaccination*

**PROG**: Free text field. Only appears if option 1 to 4 was selected in question 1.

1. What was the reason for cancelling the appointment? Multiple-choice answers are possible.

**PROG**: Multiple-choice answers are allowed. Appears only if "None, appointment cancelled" was selected in question 1.

| COVID-19 infection after vaccination | ⭘ |
| --- | --- |
| Intolerance to vaccination | ⭘ |
| Other | ⭘ |

**PROG**: Free text field. Only appears if “Other” is selected in question 2.

1. Did you receive another vaccination since the vaccination against [vaccination selected in registration]?

**PROG**: Allow only single-choice answers.

| Yes | ⭘ |
| --- | --- |
| No | ⭘ |

1. You received the following vaccination(s) afterwards. Multiple-choice answers are possible.

**PROG**: Allow multiple response options. Only appears if "Yes" was selected in question 4.

| Influenza | ⭘ |
| --- | --- |
| Shingles | ⭘ |
| Pneumococcus | ⭘ |
| COVID-19 | ⭘ |
| TBE | ⭘ |
| Tetanus (and/or diphtheria/pertussis/poliomyelitis) | ⭘ |
| None of the above | ⭘ |

1. Have you been diagnosed with COVID-19 infection since your vaccination?

**PROG**: Allow only single-choice answers.

| Yes | ⭘ |
| --- | --- |
| No | ⭘ |

Reactogenicity

1. Did you experience any reactions after the ______ vaccination? Please also record complaints that you do not attribute to the vaccination.

**PROG**: Allow only single-choice answers. The placeholder shows the vaccination(s) selected in the registration.

| Yes | ⭘ |
| --- | --- |
| No | ⭘ **PROG**: **END** |

1. Which of the following health complaints did you experience? Multiple-choice answers are possible.

**PROG**: Allow multiple-choice answers.

| Complaints at the injection site (e.g. swelling, redness, pain) | ⭘ |
| --- | --- |
| Restricted movement of the arm | ⭘ |
| Pus collection and/or abscess | ⭘ |
| Tiredness and/or fatigue | ⭘ |
| Fever > 38.0°C and/or chills | ⭘ |
| Nausea and/or vomiting | ⭘ |
| Allergic reaction (e.g. skin rash, facial swelling) | ⭘ |
| Shortness of breath | ⭘ |
| Headache | ⭘ |
| Sensory disturbance and/or numbness | ⭘ |
| Circulatory collapse | ⭘ |
| Dizziness | ⭘ |
| Seizure | ⭘ |
| Muscle or joint pain | ⭘ |
| Blood clotting disorder (e.g. thrombosis, embolism) | ⭘ |
| Other | ⭘ |

1. Which other health complaints have you experienced? Please enter your other complaints here:

**PROG**: Free text field. Only appears if "Other" was selected in question 8.

1. Have you ever had an allergic reaction to a vaccine? (e.g. skin rash, shortness of breath, swelling in the throat or face, loss of consciousness).

**PROG**: Allow only single-choice answers. Only appears if "Allergic reaction", "Shortness of breath" or "Circulatory collapse" was selected.

| Yes | ⭘ |
| --- | --- |
| No | ⭘ |
| I do not know | ⭘ |

1. Has this been your first seizure?

**PROG**: Allow only single-choice answers. Only appears if “Seizure” was selected.

| Yes | ⭘ |
| --- | --- |
| No | ⭘ |
| I do not know | ⭘ |

Consequences

1. Which consequences did your health complaints have? Multiple-choice answers are possible.

**PROG**: Allow multiple-choice answers. Only appears if at least one reaction was selected. A separate question appears for each reaction selected.

| No consequences | ⭘ |
| --- | --- |
| Medication intake | ⭘ |
| Sick leave | ⭘ |
| Outpatient (practice) consultation | ⭘ |
| Hospitalisation | ⭘ |
| Clinic (ambulant) consultation | ⭘ |

Please provide some information about your hospital stay.

1. How many times have you been taken to hospital?

**PROG**: Allow only single-choice answers. Only appears if "Hospitalisation" and/or “Clinic (ambulant) consultation” was selected.

| Once | ⭘ |
| --- | --- |
| Twice | ⭘ |
| 3 times | ⭘ |
| 4 times or more | ⭘ |

1. How long was your (longest) inpatient hospital stay in days?

**PROG**: Allow only single-choice answers. Only appears if "Hospitalisation" was selected at least once.

| 1 day | ⭘ |
| --- | --- |
| 2 days | ⭘ |
| 3 days | ⭘ |
| 4 days | ⭘ |
| 5 days | ⭘ |
| 6 days | ⭘ |
| 7 days | ⭘ |
| 8 days or more | ⭘ |

1. How did the admission(s) to the hospital take place?

**PROG**: Only appears if "Hospitalisation" and/or “Clinic (ambulant) consultation” was selected at least once.

| Referral as a planned intervention by your general practitioner or specialist | ⭘ |
| --- | --- |
| Referral as an emergency by your general practitioner or specialist | ⭘ |
| Referral by a doctor on call | ⭘ |
| Transport by the ambulance service | ⭘ |
| Own presentation in the emergency room | ⭘ |

Perception

Please tell us how you felt about your health problems.

**PROG**: Matrix fields.

1. I suspect a connection between my health problems and the vaccination.
2. I feel/felt adversely affected by the health problems.
3. My health problems will have long-term consequences for me.
4. My health problems are comparable to previous vaccinations.

**PROG**: Matrix fields. Allow only single-choice answers per question. Matrix only appears if at least one complaint has been selected.

| Strongly disagree | ⭘ |
| --- | --- |
| Disagree | ⭘ |
| Agree | ⭘ |
| Strongly agree | ⭘ |

1. With which health complaints do you suspect a connection with the vaccination? Multiple-choice answers are possible.

**PROG**: Allow multiple-choice answers. Each reaction selected appears and can be checked on if a connection with vaccination is suspected.

**End of short-term survey**

Long-term/Follow-up survey

Vaccination

1. *Added later:* Is there still an appointment planned for your 2^nd^ vaccination?

*Comment: see comment above.*

**PROG**: Allow only single-choice answers. Appears only in the long-term survey after the first vaccination and if two doses have been planned.

| Yes | ⭘ |
| --- | --- |
| No | ⭘ |

1. *Added later:* Please enter the date for your 2nd vaccination.

*Comment: see comment above.*

**PROG**: Free text field or option “no date set yet”. Appears only if “yes” has been selected in the previous question.

1. *Added later:* You received the following COVID-19 vaccine (2^nd^ dose). You will find the name of the vaccine on the label in your vaccination certificate:

**PROG**: Allow only one response option. Appears only if brand name has not been selected yet after the second vaccination.

| BNT162b2 (BioNTech/Pfizer) | ⭘ |
| --- | --- |
| mRNA-1273 (Moderna) | ⭘ |
| ChadOx1 (AstraZeneca) | ⭘ |
| Ad26.COV2.S (Johnson & Johnson) | ⭘ |
| None, appointment cancelled | ⭘ |

1. *Added later:* What was the reason for cancelling the appointment? Multiple-choice answers are possible.

**PROG**: Multiple-choice answers are allowed. Appears only if "None, appointment cancelled" was selected.

| COVID-19 infection after vaccination | ⭘ |
| --- | --- |
| Intolerance to vaccination | ⭘ |
| Other | ⭘ |

**PROG**: Free text field. Only appears if “Other” is selected.

1. Did you receive another vaccination since the last survey?

**PROG**: Allow only single-choice answers.

| Yes | ⭘ |
| --- | --- |
| No | ⭘ |

1. You received the following vaccination(s) afterwards. Multiple-choice answers are possible.

**PROG**: Allow multiple response options. Only appears if "Yes" was selected in question 1.

| Influenza | ⭘ |
| --- | --- |
| Shingles | ⭘ |
| Pneumococcus | ⭘ |
| COVID-19 | ⭘ |
| TBE | ⭘ |
| Tetanus (and/or diphtheria/pertussis/poliomyelitis) | ⭘ |
| None of the above | ⭘ |

1. Have you been diagnosed with COVID-19 infection since the last survey?

**PROG**: Allow only single-choice answers. Only appears if vaccination was against COVID-19.

| Yes | ⭘ |
| --- | --- |
| No | ⭘ |

While answering please refer to the period since the last interview (or at least 2 weeks after the last vaccination).

Medical care

1. Have you seen a doctor because of complaints? Please also refer to complaints that you do not attribute to the vaccination.

**PROG**: Allow only single-choice answers.

| Yes | ⭘ |
| --- | --- |
| Not yet, appointment planned | ⭘ |
| No | ⭘ **PROG**: **END** |

1. Have you been hospitalised due to any complaints? Please also refer to complaints that you do not attribute to the vaccination.

**PROG**: Allow only single-choice answers.

| Yes | ⭘ |
| --- | --- |
| Not yet, admission planned | ⭘ |
| No | ⭘ **PROG**: **END** |

1. Have you had one or more complaints that led to a doctor's examination and/or hospitalisation? Multiple-choice answers are possible.

**PROG**: Allow more than one answer. Only appears if "Yes" or "Not yet, appointment/admission planned" was selected.

| Headache | ⭘ |
| --- | --- |
| Dizziness | ⭘ |
| Sensory disturbance | ⭘ |
| Unconsciousness | ⭘ |
| Neuralgia | ⭘ |
| Seizure | ⭘ |
| Epilepsy | ⭘ |
| Stroke/ischemic infarction (apoplexy) | ⭘ |
| Minor stroke (TIA) | ⭘ |
| Paralysis of the facial nerve (facial paresis) | ⭘ |
| Multiple sclerosis | ⭘ |
| None of the above | ⭘ |

1. Have you had one or more complaints that led to a doctor's examination and/or hospitalisation? Multiple-choice answers are possible.

**PROG**: Allow more than one answer. Only appears if "Yes" or "Not yet, appointment/admission planned" was selected.

| Diabetes | ⭘ |
| --- | --- |
| Tachycardia/arrhythmia | ⭘ |
| Chest pain | ⭘ |
| Heart attack | ⭘ |
| Peri/myocardial infarction | ⭘ |
| Vascular inflammation (vasculitis) | ⭘ |
| Pulmonary embolism | ⭘ |
| Blood clot (thrombosis) | ⭘ |
| Coagulation disorder | ⭘ |
| None of the above | ⭘ |

1. Have you had one or more complaints that led to a doctor's examination and/or hospitalisation? Multiple-choice answers are possible.

**PROG**: Allow more than one answer. Only appears if "Yes" or "Not yet, appointment/admission planned" was selected.

| Muscle weakness | ⭘ |
| --- | --- |
| Back pain | ⭘ |
| arm, leg pain | ⭘ |
| Joint swelling | ⭘ |
| Joint inflammation (arthritis) | ⭘ |
| Muscle twitching | ⭘ |
| Movement disorder | ⭘ |
| None of the above | ⭘ |

1. Have you had one or more complaints that led to a doctor's examination and/or hospitalisation? Page 4 of 4. Multiple-choice answers are possible.

**PROG**: Allow more than one answer. Only appears if "Yes" or "Not yet, appointment/admission planned" was selected.

| Flu-like symptoms | ⭘ |
| --- | --- |
| Shortness of breath | ⭘ |
| Fever | ⭘ |
| Nausea/vomiting | ⭘ |
| Abdominal pain | ⭘ |
| Fatigue | ⭘ |
| Feeling of faintness | ⭘ |
| Feeling ill | ⭘ |
| None of the above | ⭘ |

1. If other complaints have occurred, please describe them here:

**PROG**: Free text field. Only appears if "Yes" or "Not yet, appointment/admission planned" was selected.

1. You are presented with an overview of the complaints you have indicated. Which symptoms did you already suffer from before the vaccination? Multiple-choice answers are possible.

**PROG**: Allow multiple-choice answers. All the complaints selected appear and can be checked

Perception

Please tell us how you felt about your health complaints.

**PROG**: Matrix fields.

1. I suspect a connection between my health complaints and the vaccination.
2. I feel/felt adversely affected by the health complaints.
3. My health complaints will have long-term consequences for me.
4. My health complaints are comparable to previous vaccinations.

**PROG**: Matrix fields. Allow only single-choice answers. Matrix only appears if at least one complaint has been selected.

| Strongly disagree | ⭘ |
| --- | --- |
| Disagree | ⭘ |
| Agree | ⭘ |
| Strongly agree | ⭘ |

1. With which health complaints do you suspect a connection with the vaccination? Multiple-choice answers are possible.

**PROG**: Allow multiple-choice answers. Each complaint selected appears and can be checked.

Medical report

Main diagnosis in the medical report.

1. You have almost finished. If you have a doctor's letter for the health complaints mentioned, you can enter your main diagnosis(es).

**PROG**: Free text field. Only appears if at least one complaint was selected.

**End of long-term survey**
